# Human vascularized bile duct-on-a chip: a multi-cellular micro-physiological system for studying cholestatic liver disease

**DOI:** 10.1088/1758-5090/ad0261

**Published:** 2023-10-20

**Authors:** Yu Du, Iris E M de Jong, Kapish Gupta, Orit Waisbourd-Zinman, Adi Har-Zahav, Carol J Soroka, James L Boyer, Jessica Llewellyn, Chengyang Liu, Ali Naji, William J Polacheck, Rebecca G Wells

**Affiliations:** 1 Department of Medicine, Perelman School of Medicine, University of Pennsylvania, Philadelphia, PA, United States of America; 2 Key Laboratory of Microgravity (National Microgravity Laboratory), Center of Biomechanics and Bioengineering, and Beijing Key Laboratory of Engineered Construction and Mechanobiology, Institute of Mechanics, Chinese Academy of Sciences, Beijing 100190, People’s Republic of China; 3 NSF Science and Technology Center for Engineering MechanoBiology, University of Pennsylvania, Philadelphia, PA, United States of America; 4 Institute for Gastroenterology, Nutrition and Liver Diseases, Schneider Children’s Medical Center of Israel, Petach Tikva, Israel; 5 Sackler Faculty of Medicine, Tel-Aviv University, Tel-Aviv, Israel; 6 Department of Internal Medicine, Section of Digestive Diseases and Liver Center, Yale University School of Medicine, New Haven, CT, United States of America; 7 Department of Surgery, University of Pennsylvania, Philadelphia, PA, United States of America; 8 Joint Department of Biomedical Engineering, University of North Carolina at Chapel Hill and North Carolina State University, Chapel Hill, NC, United States of America; 9 Department of Bioengineering, School of Engineering and Applied Sciences, University of Pennsylvania, Philadelphia, PA, United States of America; 10 Department of Pathology and Laboratory Medicine, Perelman School of Medicine at the University of Pennsylvania, Philadelphia, PA, United States of America

**Keywords:** microfluidic device, cholangiopathy, tissue engineering, organ-on-a-chip, primary sclerosing cholangitis

## Abstract

Exploring the pathogenesis of and developing therapies for cholestatic liver diseases such as primary sclerosing cholangitis (PSC) remains challenging, partly due to a paucity of *in vitro* models that capture the complex environments contributing to disease progression and partly due to difficulty in obtaining cholangiocytes. Here we report the development of a human vascularized bile duct-on-a-chip (VBDOC) that uses cholangiocyte organoids derived from normal bile duct tissue and human vascular endothelial cells to model bile ducts and blood vessels structurally and functionally in three dimensions. Cholangiocytes in the duct polarized, formed mature tight junctions and had permeability properties comparable to those measured in *ex vivo* systems. The flow of blood and bile was modeled by perfusion of the cell-lined channels, and cholangiocytes and endothelial cells displayed differential responses to flow. We also showed that the device can be constructed with biliary organoids from cells isolated from both bile duct tissue and the bile of PSC patients. Cholangiocytes in the duct became more inflammatory under the stimulation of IL-17A, which induced peripheral blood mononuclear cells and differentiated Th17 cells to transmigrate across the vascular channel. In sum, this human VBDOC recapitulated the vascular-biliary interface structurally and functionally and represents a novel multicellular platform to study inflammatory and fibrotic cholestatic liver diseases.

**Abbreviations**
PSC: Primary sclerosing cholangitis
VBDOC: Vascularized bile duct-on-a-chip
ERCP: Endoscopic retrograde cholangiopancreatography
CCCM: Complete cholangiocyte culture medium
EGM: Endothelial growth medium
HUVEC: Human umbilical vein endothelial cell
ECM: Extracellular matrix
PDMS: Polydimethylsiloxane
FITC: Fluorescein isothiocyanate
PBS: Phosphate buffered saline
Pd: Permeability coefficient
PFA: Paraformaldehyde
BSA: Bovine serum albumin
DAPI: 4′,6-diamidino-2-phenylindole
ZO-1: Zona occludens 1
MDR1: Multidrug resistance protein-1
ASBT: Apical sodium-dependent bile salt transporter
CFTR: Cystic fibrosis transmembrane conductance regulator
SCTR: Secretin receptor
ICAM-1: Intercellular cell adhesion molecule-1
VCAM-1: Vascular cell adhesion molecule-1
LPS: Lipopolysaccharide
PBMC: Peripheral blood mononuclear cell

## 1. Introduction

Although the pathogenesis of cholestatic liver diseases such as PSC remains poorly understood [1, 2], evidence suggests that disease evolution involves cells at the vascular-biliary interface, including cholangiocytes (the cells that line the bile ducts), immune cells, endothelial cells, and mesenchymal cells [3–5]. PSC results in inflammation, scarring, and eventual blockage of both large and small bile ducts, ultimately causing damage to the liver itself, with fibrosis and cirrhosis. The multifactorial etiology, heterogeneity and complex surrounding microenvironment of PSC and other cholestatic liver diseases makes them difficult to model [4] due to the challenges required to capture multiple different features of these diseases (including multicellular interactions, fibrosis, and tubular structures) and to incorporate mechanical stimuli, easy cell access and manipulation of various parameters. Animal models such as *Abcb4^-/-^* mice, lithocholic acid-fed mice and *Tgr5^-/-^* mice [6–8] incorporate all relevant cell types and capture their interactions in a physiological environment; however, due to inter-species variations (potentially including the composition of bile and the gut microbiome), mouse models still only partially reproduce the phenotype of human cholangiopathies.

On the other hand, organoids derived from primary human cells have the advantage that the cells appear to maintain their original phenotypes [9]; such organoids have now been developed to model cholangiopathies including biliary atresia, Alagille Syndrome, and PSC [9–11]. While these avoid many problems associated with rodent systems, they have a non-tubular 3D structure, lack flow and are hard to model as multicellular systems. A third category of model system, microfluidic organs-on-chips, offers the potential to incorporate 3D structure, multiple cell types, and mechanical stimuli [12]. The development of a bile duct-on-a-chip featuring a tubular duct that exhibits normal cholangiocyte apical-basal polarity, barrier function and physiological responses to applied flow was previously reported [13]. Organs-on-chips, however, face the challenges of cell source and cellular fidelity; this is particularly problematic for a rare disease like PSC. Integrating these latter two methods—organoids and organs-on-chips—offers a way to bypass the limitations of each and to provide an *in vitro* approach capable of advancing the study of cholangiopathies [14].

We report here the development of a novel multicellular *in vitro* platform, a human VBDOC incorporating cholangiocytes from control and PSC patients as well as human fibroblasts and endothelial cells. We used the VBDOC to recapitulate the barrier functions of normal and diseased biliary blood vessels and bile ducts, test their responses to mechanical force (flow), and study cytokine secretion and immune cell transmigration in response to inflammatory stimuli. The human VBDOC represents a novel multicellular *in vitro* platform to study the pathophysiology of the biliary system using cholangiocytes from a variety of sources.

## 2. Materials and methods

### 2.1. Isolation

Extrahepatic bile ducts were obtained from previously-healthy deceased organ donors (one 51-year-old female, one 66-year-old male) as part of the Human Pancreas Procurement and Analysis Program at the University of Pennsylvania, which was granted an IRB exemption (protocol 826 489). A bile duct from a 40-year-old male PSC patient was obtained at surgery, with ethics approval granted by the Beilinson Medical Ethics Committee (approval number: 0072-19-RMC). Cholangiocytes were isolated by mechanically scraping the luminal layer of these bile ducts, according to a protocol described previously [15]. Bile-derived cholangiocyte-like cells were isolated from bile collected at ERCP as described [11, 16] at the Yale New Haven Hospital, with an IRB exemption (protocol 2000 020 797). Research was conducted in accordance with the principles embodied in the Declaration of Helsinki. All samples were anonymized and informed consent was obtained from patients or next of kin in accordance with the requirements of the local IRB.

### 2.2. Generation and culture of organoids

Organoids were cultured as previously described [15]. Primary cholangiocytes were centrifuged at 444 g for 4 min and resuspended in a mixture of 66% Matrigel (BD Biosciences, Franklin Lakes, NJ, USA) and 33% CCCM (William’s E medium (Gibco, Life Technologies, OK, USA) supplemented with 10 mm nicotinamide, 17 mm sodium bicarbonate, 0.2 mm 2-phospho-l-ascorbic acid trisodium salt, and 14 mm glucose (all from Sigma-Aldrich, St. Louis, MO; chemicals unless noted are all from Sigma-Aldrich); 6.3 mm sodium pyruvate, 20 mm 4-(2-hydroxyethyl)-1-piperazineethanesulfonic acid (HEPES) and 2 mM Glutamax (Invitrogen, Carlsbad, CA, USA); ITS + premix (BD Biosciences), 100 U ml^−1^ penicillin and 100 *μ*g ml^−1^ streptomycin (Thermo Fisher Scientific, Waltham, MA, USA); and 0.1 *μ*m dexamethasone 20 ng ml^−1^ EGF, 500 ng ml^−1^ R-spondin and 100 ng ml^−1^ DKK-1 (R&D Systems, Minneapolis, MN, USA) with 10 *μ*m Y27632. The cell suspension was plated in a 24-well plate at 300 *μ*l well^−1^ (∼1 × 10^5^ cells) and plates were then incubated at 37 °C for 30 min until the Matrigel solidified. Subsequently, 1 ml of CCCM with 10 *μ*m Y27632 was added to each well. The culture medium was switched to CCCM alone after 2 d and changed every 2 d thereafter.

### 2.3. Culture and transfection of human gall bladder fibroblasts

Primary human gallbladder fibroblasts were purchased from ScienCell (5430, Carlsbad, CA, USA), and cultured using the fibroblast growth medium kit from Lonza (CC-3132; Los Angeles, CA, USA). Green fluorescent protein (GFP)-labeled cells were generated by infecting them with GFP lentivirus (17 448; Addgene, Watertown, MA, USA).

### 2.4. Culture of HUVECs

HUVECs (Lonza, CC-2519) were cultured using the EGM kit from Lonza (CC-3202) according to the supplier’s instructions. HUVECs under passage 7 were used for all experiments.

### 2.5. Fabrication of the VBDOC

#### 2.5.1. PDMS device preparation

Microfluidic molds were fabricated using high-resolution 3D printing by Proto Labs, Inc. (Maple Plain, MN, USA). PDMS (Sylgard 184, Dow-Corning, Midland, MI, USA) devices were cast by pouring the PDMS mixture of base and curing agent (weight ratio 10:1) into the mold, followed by degassing and full cure at 75 °C for 1.5 h. Cured PDMS gels were cut into bricks of equal size (2 cm × 1.5 cm). Ports were punched and the device was cleaned with tape. The PDMS was bonded to the coverslip by 45 s of plasma treatment. The collagen gel chamber was treated with 0.01% (v/v) poly-L-lysine (about 80 *μ*l per device) for 1 h and 0.5% (v/v) glutaraldehyde (about 80 *μ*l per device; Thermo Fisher Scientific) for 20 min at room temperature to promote collagen adhesion to PDMS. After the devices were washed overnight in water and for 30 min in 70% ethanol, steel acupuncture needles (200 *μ*m diameter; Seirin, Kyoto, Japan) were inserted from opposite directions though the channels (figure S1) and the devices were then sterilized under UV for 20 min.

#### 2.5.2. Cell seeding and culture

Neutralized collagen solution (2.5 mg ml^−1^, pH = 7.0) was prepared by mixing rat tail type 1 collagen (Thermo Fisher Scientific), 10X DMEM medium, 10 mm HEPES, 1 m NaOH and NaHCO_3_ (0.035% w/v) on ice. Immediately after collagen gel preparation, fibroblasts were prepared by trypsinizing them from culture dishes, then washing and resuspending them with the neutralized collagen solution at a density of 1.5 × 10^5^ ml^−1^. The fibroblast/collagen mixture was injected through the side ports of the device to fill the collagen gel chamber (about 70 *μ*l per device) and the collagen was allowed to solidify by incubating the devices inverted at 37 °C for 20 min. After the collagen gel solidified, all ports were filled with fibroblast growth medium (about 120 *μ*l per reservoir port) and incubated overnight, then needles were removed to yield two parallel collagen channels and the channel ends at the edges of the PDMS bricks were sealed with vacuum grease to prevent medium leakage.

A suspension of 5 × 10^5^ ml^−1^ of cholangiocytes was introduced into the reservoir ports (40 *μ*l in one port and 30 *μ*l in the other) connected to the cholangiocyte channel. Cells were allowed to adhere to the top surface of the channel for 5 min; devices were then flipped to allow cells to adhere to the bottom surface of the channel for 5 min. (Times and cell amounts were determined empirically to yield an even cell coating in the channel with sufficient adhesion to permit rinsing the channel.) Nonadherent cells were removed by rinsing with cell-culture medium, and the devices were filled with fresh CCCM with 10 *μ*m Y27632 (about 120 *μ*l per reservoir port). Devices were maintained at 37 °C (5% CO_2_) on a rocker at 5 rpm for 2 d. The medium was switched to CCCM with medium changes every 2 d until the development of confluent monolayers.

When the cholangiocyte monolayer was just confluent, endothelial cells were prepared by trypsinizing HUVECs from culture dishes, then washing and resuspending them in EGM at a density of 5 × 10^5^ ml^−1^. The cell suspension was introduced to the reservoir ports (40 *μ*l in one port and 30 *μ*l in the other) connected to the endothelial channel. Cells were allowed to adhere to the top surface of the channel for 2 min; devices were then flipped to allow cells to adhere to the bottom surface of the channel for 2 min. (Times and cell numbers were again determined empirically.) Nonadherent cells were removed by rinsing with EGM. The reservoir ports connected to the endothelial channel were filled with EGM while the reservoir ports connected to the cholangiocyte channel were filled with CCCM (about 120 *μ*l per reservoir port). Devices were maintained at 37 °C (5% CO_2_) on a rocker at 5 rpm (static groups were maintained at 37 °C (5% CO_2_) on flat incubator shelf) until the development of confluent endothelial monolayers and compact cholangiocyte monolayers [13].

### 2.6. Permeability measurements

To measure the permeability of the cholangiocyte and endothelial cell monolayers in the channels, fluorescent dextran (70, 10, and 4 kDa, labeled with FITC) in PBS was introduced into the channels at a concentration of 20 *μ*g ml^−1^. Diffusion of the dextran was imaged in real time with an EVOS FL Auto 2 Imaging System (Thermo Fisher Scientific) at 10× magnification. The diffusive permeability coefficient was calculated by measuring the flux of dextran into the collagen gel and fitting the resulting diffusion profiles to a dynamic mass conservation equation, as described [13, 17].

### 2.7. Immunostaining

Cholangiocyte and endothelial cell monolayers in the device were fixed with 4% PFA (Thermo Fisher Scientific) at 37 °C for 20 min with rocking. Cells were rinsed 3× with PBS and permeabilized with 0.1% Triton X-100 for 3 d, then blocked with 2% BSA in PBS at 4 °C overnight with rocking. Primary antibodies, together with DAPI (Thermo Fisher Scientific) diluted in 2% BSA in PBS, were incubated overnight at 4 °C and rinsed 3× with PBS for 5 min each with rocking, followed by an overnight rinse. Secondary antibodies were diluted in 2% BSA in PBS, incubated overnight at 4 °C, and rinsed 3× with PBS for 5 min each on a rocker. Primary antibodies and the concentrations used are listed in supporting table S1. Cyanine (Cy)3- and Cy5-conjugated secondary antibodies were used at 1:400 (Vector Laboratories, Burlingame, CA, USA).

### 2.8. Cytokine array

Cholangiocytes in the devices were cultured in CCCM with or without IL-17A for 48 h, after which the media in all four reservoirs was collected and analyzed with the Human Cytokine Antibody Array (Abcam, Cambridge, UK). The full list of cytokines tested in the kit is found in supporting tables S2 and S3.

### 2.9. Rhodamine 123 transport assay

Rhodamine 123 transport assays were performed in the VBDOC with only cholangiocytes in the device. The 5 *μ*m Rhodamine 123 (Thermo Fisher Scientific) in CCCM was perfused to the basal side through the endothelial channel (without endothelial cells), resulting in it diffusing through the whole chamber, and normal CCCM was added to the cholangiocyte channel. The cholangiocyte channel ends were then blocked with vacuum grease to ensure that there was no Rhodamine 123 in the lumen at the start of the assay and to avoid Rhodamine 123 dilution by medium in the reservoir ports. After incubating for 2 h at 37 °C, the basal side was washed 3× with PBS via the endothelial channel.

In the inhibition assay, cholangiocytes were incubated with 10 *μ*m Verapamil in CCCM at 37 °C for 30 min, followed by incubation with Rhodamine 123, as described above. Images were acquired using a Leica confocal microscope and Leica application suite (LAS X; Leica, Buffalo Grove, IL, USA). Fluorescence intensity was measured across the cholangiocyte channel.

### 2.10. *In vitro* Th17 differentiation

CD4 T cells were provided by the Human Immune Core at the University of Pennsylvania. They were cultured on anti-CD3 (5 *μ*g ml^−1^ in PBS, overnight at 4 °C)-coated plates in complete Roswell Park Memorial Institute (RPMI) (RPMI1640 + Glutamax medium, supplemented with 10% fetal calf serum (FCS) and 1% P/S). For Th17 differentiation, CD4 T cells were plated at a density of 2 × 10^6^ cells per well (24 well plates) with 50 ng ml^−1^ of human IL23 (Miltenyi, Germany) and IL1b (Miltenyi) in 500 *μ*l complete RPMI. On day 4, medium and cytokine mix were replaced. On day 7, cells were collected for transmigration assays and Th17 levels were analyzed by flow cytometry.

### 2.11. Transmigration assay

After cholangiocytes and endothelial cells reached confluence in their individual channels in the VBDOC, the cholangiocytes were treated with 50 ng ml^−1^ IL-17A in culture medium for 48 h; for the last 4 h, the endothelial cells were also treated with 1 *μ*g ml^−1^ LPS. PBMCs provided by the Human Immune Core of University of Pennsylvania or differentiated CD4 T cells were fluorescently labeled in RPMI with 5 *μ*m Celltracker Green CMFDA (Thermo Fisher Scientific) for 30 min at 37 °C and resuspended at 2 × 10^6^ ml^−1^ in EGM after washing with EGM. The 110 *μ*l of the cell suspension was added to one side of the reservoir port, 90 *μ*l was added to the other side of the reservoir port, and the devices were placed in the incubator overnight. Images of transmigrated cells in the VBDOC were taken by confocal z-stack scanning.

### 2.12. Statistical analysis

Statistical significance was assessed using the Student’s *t*-test with equal variance if they passed the normality test (Shapiro–Will) and the Mann–Whitney test if not for two groups and using one-way ANOVA test if they passed the normality test and ANOVA on RANKs if not for multiple groups. *p* < 0.05 was regarded as statistically significant and calculated with Prism 7 (GraphPad Software, La Jolla, CA, USA) and SigmaPlot 12.5 (San Jose, CA, USA). All data are presented as mean ± standard deviation (SD). Sample size is indicated in the figure legends.

## 3. Results

### 3.1. Fabrication of the VBDOC

**Figure 1.**
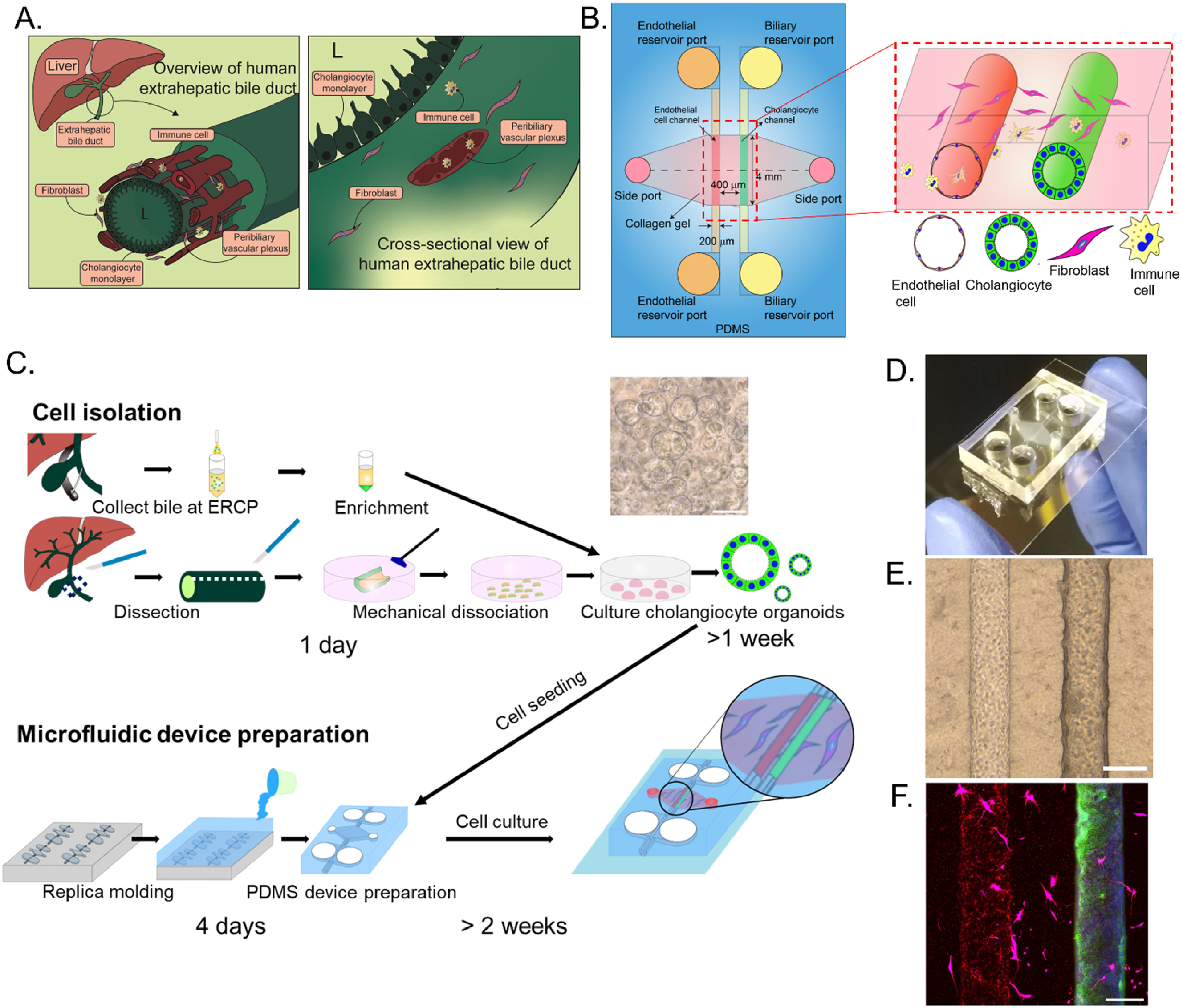
Fabrication of a human VBDOC using organoid-derived cholangiocytes. (A). Schematic showing the anatomic structure of the extrahepatic bile ducts, in particular the relationship between the vasculature, fibroblasts, and cholangiocytes. (B). Schematic showing top and cross-sectional views of the VBDOC. (C). Flow chart showing an overview of the process of cell isolation, microfluidic device preparation and device seeding. (D). Image of a typical VBDOC, top view. (E). Representative bright field image of the endothelial (left) and biliary (right) channels with fibroblasts in the surrounding matrix. (F). Representative immunofluorescence images of endothelial cells (VE-cadherin, red), fibroblasts (transfected GFP, magenta) and cholangiocytes (K19, green) in the VBDOC. Nuclei shown by DAPI staining (blue). Each image is representative of at least three independently-constructed and seeded devices for each condition. Scale bars: 200 *μ*m.

In order to recapitulate the vascular-biliary-mesenchymal interface in three dimensions, which is believed to be important in the development of cholangiopathies, we designed a two-channel microfluidic device modified from a previously-described model of sprouting angiogenesis [18]. To generate two channels (a vascular channel and a bile duct) with an intervening submucosa-like region, a collagen solution containing human gallbladder fibroblasts was injected through the side ports of a PDMS device (figures 1(A), (B) and S1) and gelled around two parallel needles (200 *μ*m diameter) inserted through the chamber in the middle of the device. The two channels formed after removing the needles were connected to two separate pairs of reservoirs.

To optimize the phenotype of the cholangiocytes in the VBDOC, we generated human cholangiocyte organoids using primary cells derived from normal tissue. Cholangiocytes from human EHBD were isolated, cultured and expanded as organoids in Matrigel with cholangiocyte medium as described previously [11, 15]. These cells were then seeded into one channel and underwent self-organization into a confluent and then compact epithelial monolayer after about two weeks [13]. As soon as the cholangiocyte monolayer became confluent, endothelial cells were seeded in the other channel, developing into a confluent monolayer after 3 d (figure 1(D)). The vascular channel, bile duct channel and surrounding mesenchymal cells in the device (figure 1) maintained structural integrity for at least a week as assessed by morphology under a bright field microscope.

### 3.2. Characterization of normal cholangiocytes in the device

**Figure 2.**
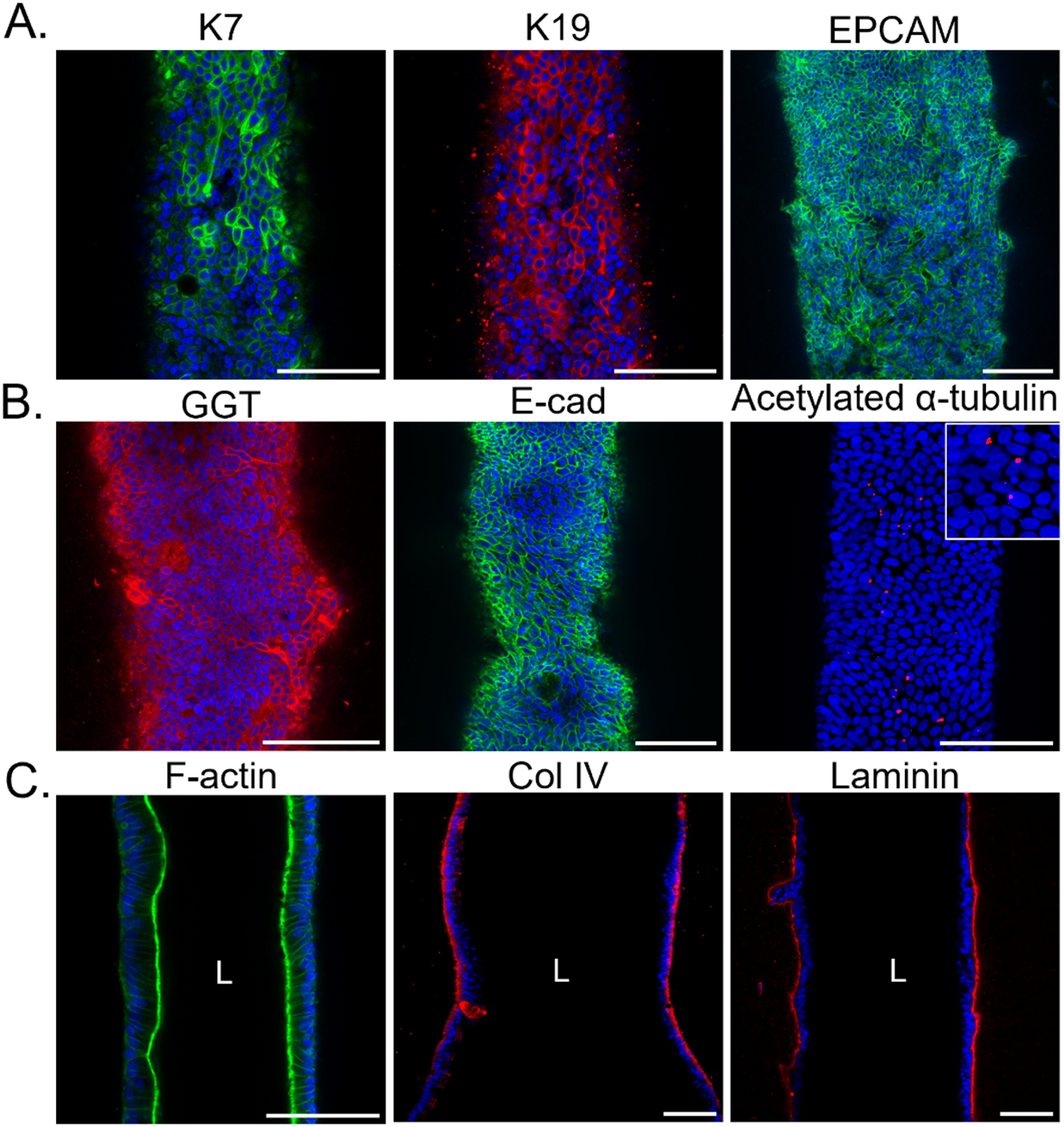
Characterization of cholangiocytes in channels. Channels lined with normal cholangiocytes derived from control tissue. Representative confocal images of cholangiocytes in the device stained with antibodies against (A) K7 and K19 (same sample stained with two antibodies), and EPCAM, (B) GGT, E-cadherin, and acetylated α-tubulin (top projection view with magnified inset), (C) F-actin, collagen IV, and laminin (mid-longitudinal cross-sectional view). Nuclei shown by DAPI staining (blue). Each image is representative of at least three independently-constructed and seeded devices. Scale bars: 100 *μ*m.

We generated cholangiocyte-lined channels successfully using cells from dissociated organoids derived from normal tissue (figure 1), then characterized them with immunofluorescence staining. Cells maintained expression of the cholangiocyte markers K7, K19, EPCAM, GGT and E-cadherin (figures 1(F) and 2). Apical F-actin and primary cilia with basal collagen IV and laminin (produced by the cholangiocytes themselves, since there was none coating the channel) demonstrated in mid-longitudinal cross-section images that cholangiocytes in the device were polarized (figure 2). Note that the channel diameter is uniform at the time of device construction, but irregularities may develop during cell culture due to cell contractility or cell-mediated matrix degradation.

### 3.3. Barrier function and bile salt transport activity of the biliary channel in the device

**Figure 3.**
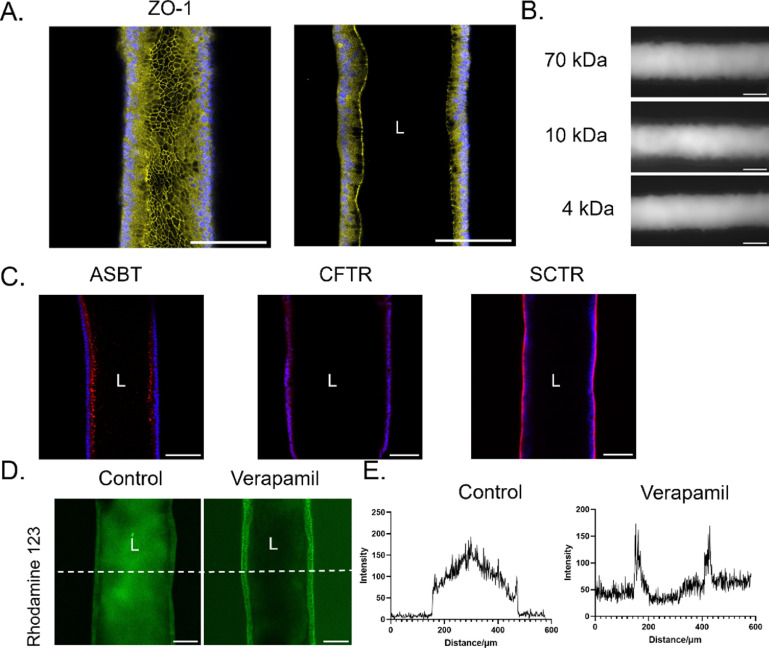
Functional characterization of cholangiocytes in the VBDOC. Channels lined with normal cholangiocytes derived from control tissue. (A). Immunofluorescence images of the channel stained with antibodies against ZO-1 (left panel: top projection view, right panel: middle cross-sectional view). Nuclei shown by DAPI staining (blue). Scale bars: 100 *μ*m. (B). Representative fluorescent images of FITC-dextran (70 kDa, 10 kDa and 4 kDa) diffusion after 2 min in the cholangiocyte channels of the VBDOC. Scale bar: 200 *μ*m. (C). Immunofluorescence images of the channel stained with antibodies against ASBT, CFTR and SCTR (middle cross-sectional view). Nuclei shown by DAPI staining (blue). Scale bars: 100 *μ*m. (D). Fluorescent rhodamine 123, a substrate of MDR1, was taken up from the basal side then secreted into the lumen of the cholangiocyte channel (left), with inhibition by the MDR1 inhibitor verapamil (right) (middle cross-sectional view). (E). Graphs depict the fluorescence intensity (from (D)) along the dotted white lines in the images. Each image is representative of at least three independently-constructed and seeded devices for each condition.

**Figure 4.**
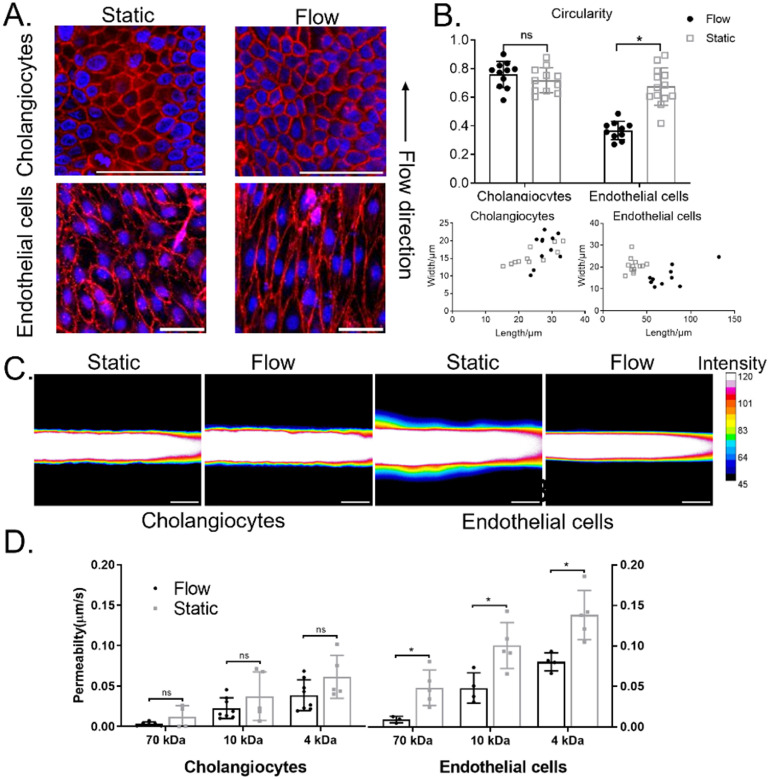
Morphological and functional responses of endothelial cells and control cholangiocytes to luminal flow. (A). Representative confocal images of cholangiocytes stained with F-actin and endothelial cells stained with VE-cadherin under static or flow conditions in the VBDOC. Nuclei shown by DAPI staining (blue). Each image is representative of at least three independently-constructed devices for each condition. Scale bars: 50 *μ*m. (B). Graphs quantifying length vs. width (lower panel) and circularity (upper panel) in endothelial cells and cholangiocytes under static or flow conditions in the VBDOC. (C). Representative pseudo-colored images (fluorescent intensity displayed in colors according to the royal look up table in ImageJ) of FITC-dextran (70 kDa) diffusion after 2 min in the cholangiocyte and endothelial channels of the VBDOC under static and flow conditions. Scale bar: 200 *μ*m. D. Permeability of the cholangiocyte channel and endothelial channel to FITC-dextran (70, 10, 4 kDa) under static and flow conditions, *n* = 4–8.

**Figure 5.**
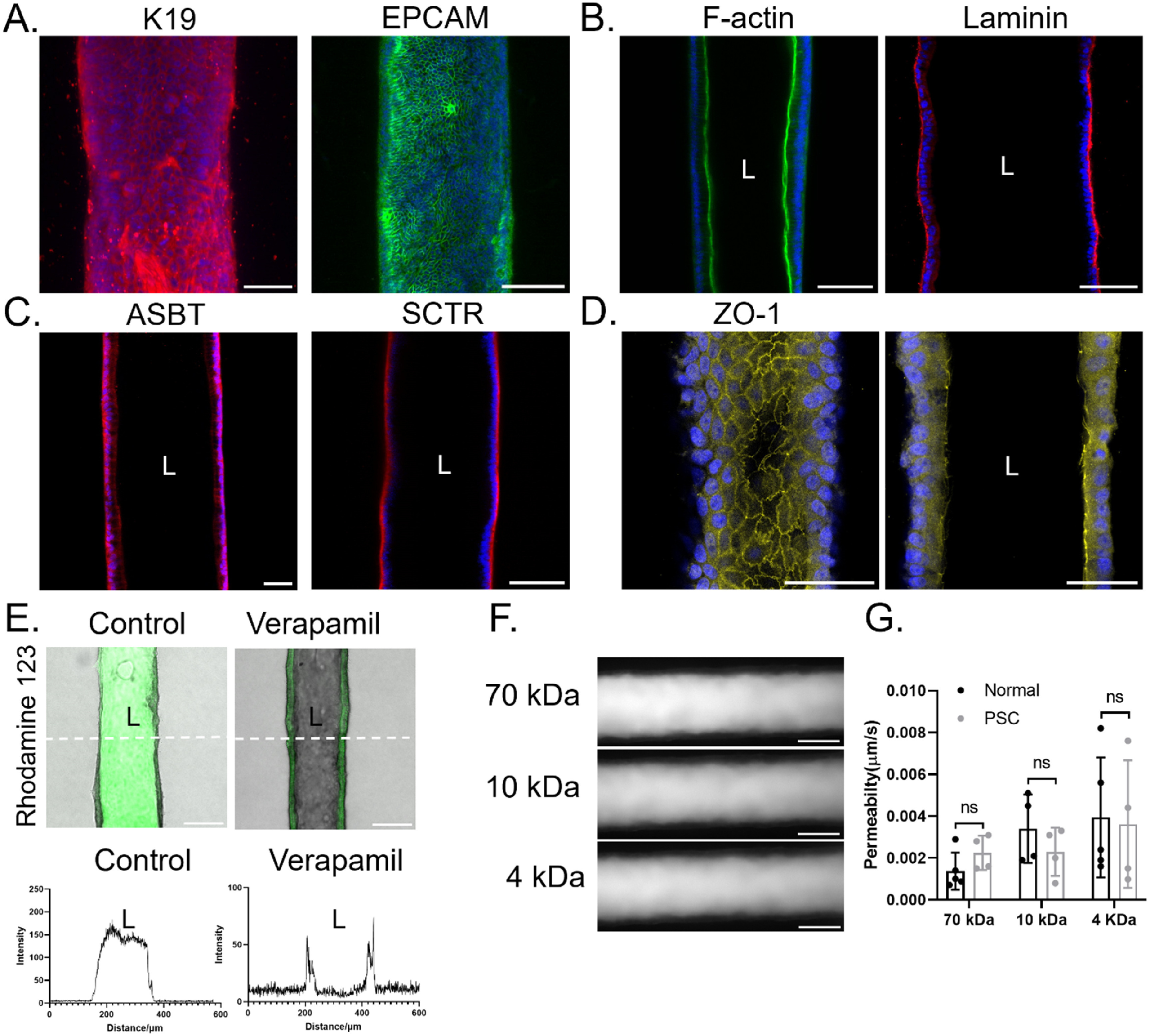
Expression and functional characterization of PSC cholangiocytes from tissue lining the biliary channel. Immunofluorescence images of PSC cholangiocytes stained with antibodies against (A) K19 and EPCAM (top projection view; note that the K19 staining was done simultaneously with K7 staining, which is shown in figure S2), (B) F-actin and laminin (middle cross-sectional view), (C) ASBT, SCTR (middle cross-sectional view), (D) ZO-1 (left panel: top projection view, right panel: middle cross-sectional view). Nuclei shown by DAPI staining (blue). Scale bars: 100 *μ*m. (E). Bright field and fluorescent images showing secretion of fluorescent rhodamine 123 (green), a substrate of MDR1, into the lumen of the cholangiocyte channel (left), with inhibition by the MDR1 inhibitor verapamil (right). Scale bars: 200 *μ*m. (F). Graphs depict the fluorescence intensity along the dotted white lines in the images. (G). Representative images of FITC-dextran (70 kDa) diffusion after 2 min in the cholangiocyte channels of the VBDOC. Scale bar: 200 *μ*m. (H). Quantification of permeability of the control and PSC cholangiocyte-lined channels to FITC-dextran (70 kDa, 10 kDa and 4 kDa), *n* = 4–5 devices, each device tested sequentially with FITC-dextran from 70 to 4 kDa. All data are presented as mean ± SD, *P < 0.05. Each image is representative of at least three independently-constructed devices for each condition.

We next examined the expression of ZO-1 by cholangiocytes in the devices by using immunofluorescence staining. The continuous and apical expression of ZO-1 at cell-cell junctions confirmed the formation of tight junctions (figure 3(A)). To examine the barrier function of the cholangiocyte-seeded channels, we perfused them with FITC-dextran ranging in size from 4 to 70 kDa. There was no obvious leakage of fluorescent dextran of any size into the collagen matrix even after 10 min (figure 3(B)), with permeability (0.04 *μ*m s^−1^ for 4 kDa dextran, figure 5(G)) comparable to *ex vivo* measurements (0.45 *μ*m s^−1^ for inulin in rat bile duct [19], 0.01 *μ*m s^−1^ for inulin in guinea pig bile duct [20], 0.03 *μ*m s^−1^ for 4 kDa dextran in a single channel bile duct-on-a-chip generated using murine cholangiocytes [13]) (figure 4(D)).

We examined the bile salt transporter expression of cholangiocytes in the VBDOC by immunostaining for the apical sodium-dependent bile acid transporter (SLC10A2; also known as ASBT), the CFTR and the basal SCTR (figure 3(C)). Transporter activity was interrogated by examining the ability of cholangiocytes to transport rhodamine 123 (figures 3(D) and (E)). Confocal microscopy showed that cholangiocytes lining the channel took up rhodamine 123 from their basal side and secreted it into the lumen and that this luminal extrusion of rhodamine 123 was inhibited by the MDR1 inhibitor verapamil, thereby confirming active transport through MDR1. Our data thus demonstrate that cholangiocytes in the VBDOC maintain functional properties in the devices.

### 3.4. Different mechanosensitivity of cholangiocytes and endothelial cells to shear flow

In order to define the flow sensitivity of cholangiocytes compared to endothelial cells, the different cells were co-cultured in neighboring channels and the devices were placed on a rocker. The rocking causes oscillating flow through the channels [17, 21], enabling us to examine the responses of cholangiocytes and endothelial cells to shear flow. We used F-actin and VE-cadherin immunostaining to assay for morphological changes. Cholangiocytes maintained a similar cuboidal shape and random alignment under both static and flow conditions. Endothelial cells, however, became elongated under flow and aligned with the direction of flow (figures 4(A) and (B)). Because barrier function is crucial for both vascular and biliary function, we compared the permeability under static and flow conditions for both kinds of cells (figures 4(C) and (D)). Consistent with data reported previously, flow promotes the establishment of a functional vascular barrier in the endothelial channel [17], as shown by decreased permeability. In contrast, the permeability of cholangiocyte monolayers remained constant independent of shear flow.

### 3.5. Construction of functional VBDOCs with organoids from PSC patients

To demonstrate that the VBDOC is applicable to the study of cholestatic liver diseases such as PSC, we seeded the device using cholangiocyte organoids derived from PSC patient tissue. The cholangiocyte markers K7, K19, GGT, EPCAM and E-cadherin (figures 5(A) and S1); the polarity markers F-actin (apical) and acetylated *α*-tubulin (primary cilia; apical), collagen IV (basal) and laminin (basal; figures 5(B) and S2); the bile salt transporters ASBT, SCTR, figure 5 (C); and the tight junction component ZO-1, figure 5 (D) were expressed in expected locations in PSC cholangiocytes in the VBDOC. Similar to normal cholangiocytes, CFTR expression was low in the PSC cholangiocytes (figure S2; compare to figure 3(C)). Bile transport activity was demonstrated by luminal extrusion of rhodamine 123 that was inhibited by the MDR1 inhibitor verapamil (figure 5(E)). Barrier function was demonstrated by perfusing FITC-Dextran ranging in size from 4 to 70 kDa; there was no obvious leakage of fluorescent dextran of any size into the collagen matrix even after 10 min (figure 5(F)). Quantification of permeability showed no significant difference between normal and PSC cholangiocyte channels (figure 5(G)).

To broaden the cell source, especially from PSC patients, we also seeded cholangiocyte-like cells dissociated from bile-derived organoids from a PSC patient into the devices [11]. The bile-derived cells, like those from tissue, survived and generated confluent monolayers in the devices. Immunofluorescence staining for K7, K19, GGT, EPCAM, F-actin, collagen IV, laminin, and acetylated *α*-tubulin generally showed similar expression of biliary markers and polarity as observed for tissue-derived cholangiocytes, although ASBT and laminin staining showed more discontinuities (figure S3).

### 3.6. Cytokine profile of normal and PSC cholangiocytes in the VBDOC with IL-17A stimulation

**Figure 6.**
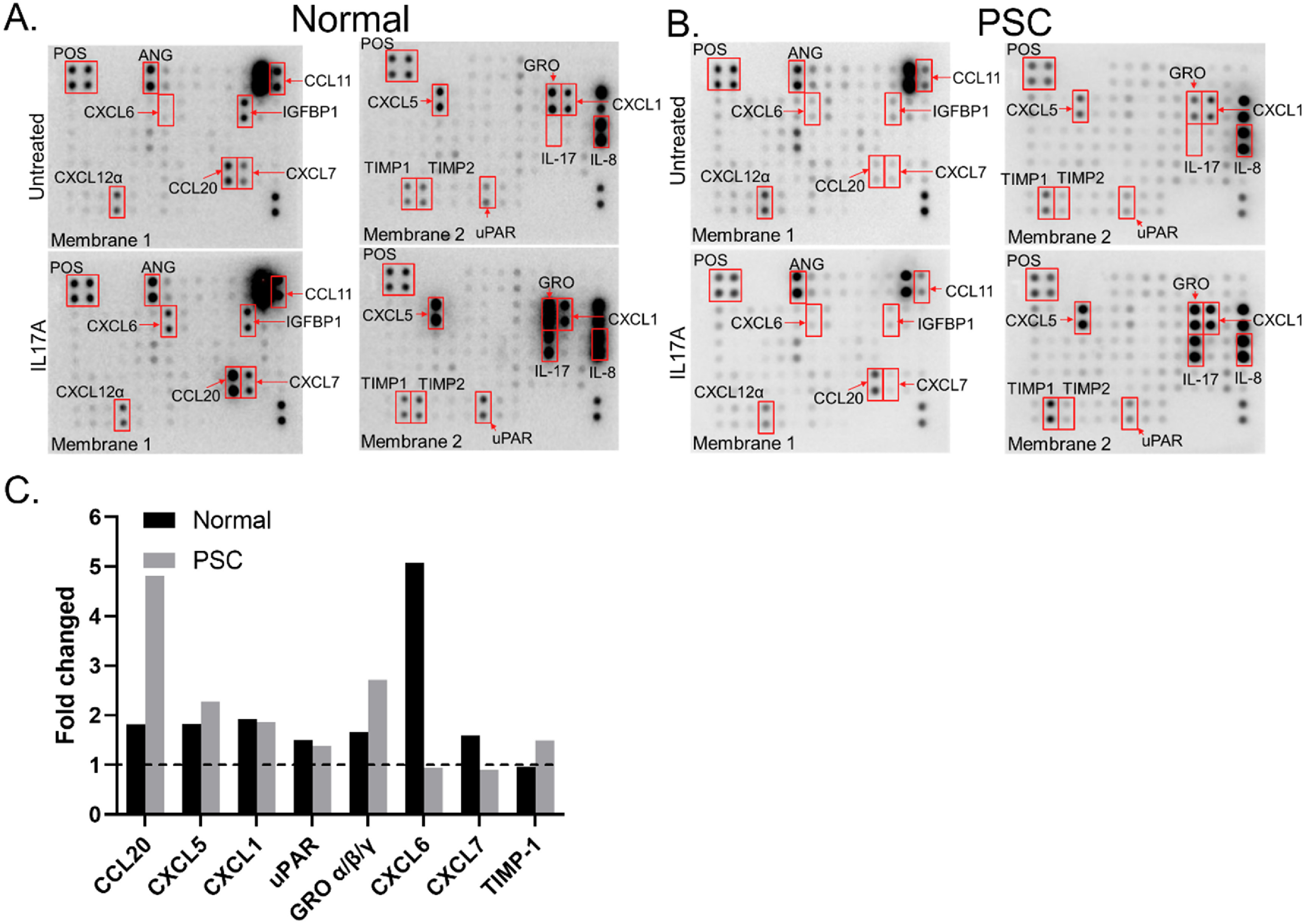
Cytokine profile of normal and PSC cholangiocytes in the VBDOC after stimulation with IL-17A. (A), (B). Human cytokine array of untreated (upper panels) and IL-17A treated (lower panels) control (A) and PSC (B) cholangiocytes in the biliary channel. (C). Quantification of the blots, showing fold change in protein expression normalized to untreated samples.

Cholangiocytes can react to inflammation and liver damage by acquiring a reactive inflammatory phenotype in which they secrete proinflammatory and profibrotic chemokines and cytokines [3, 19]. We tested whether control and PSC tissue cholangiocytes in the devices could be stimulated to secrete chemokines. Based on reports that IL-17 can stimulate biliary epithelial cells to secrete CCL20 [11, 20], we incubated cholangiocytes with IL-17A (50 ng ml^−1^ for 48 h) and measured the cytokines secreted into the culture supernatant. Normal and PSC cholangiocytes expressed similar amounts of IL-8 and angiogenin with and without IL-17A stimulation, but demonstrated increased expression of CCL20, CXCL5, CXCL1, uPAR, and GRO *α*/*β*/*γ*. In response to IL-17A, normal cholangiocytes also increased expression of CXCL6 and CXCL7, while PSC cholangiocytes increased expression of TIMP-1 (figure 6). (PSC biliary-like cells from bile-derived organoids exhibited a similar inflammatory profile as PSC cholangiocytes from tissue-derived organoids, figure S4.) By collecting the small volume of medium in reservoirs connected to the lumens of the chips, we showed that cholangiocytes in the devices can be stimulated to react in an inflammatory manner, including secreting CCL20, which has been studied previously [11, 20].

### 3.7. Immune responses in the VBDOC in response to IL-17A stimulation

**Figure 7.**
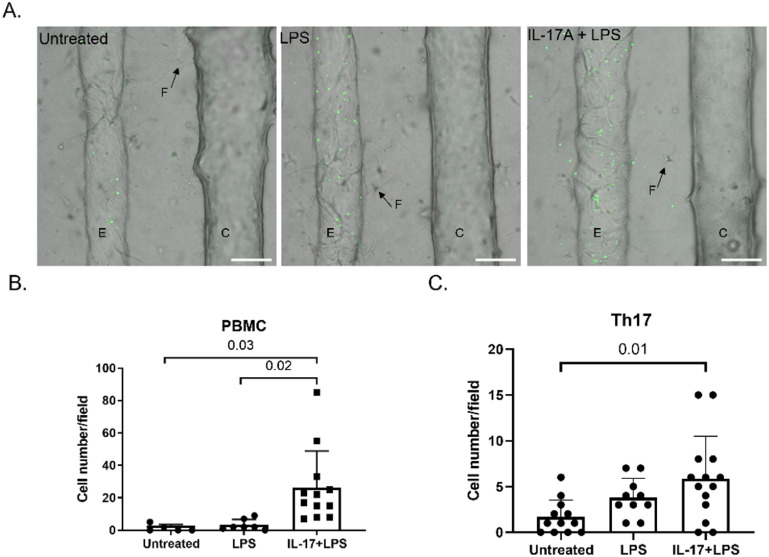
Immune cell transmigration in the VBDOC. (A). Representative images of transmigration of cell tracker-labeled (green) immune cells through the endothelium when treated with vehicle, LPS, or LPS combined with cholangiocyte IL-17A stimulation. (E): endothelial cells, (C): cholangiocytes, (F): fibroblasts (representative cells shown by arrows). (B). Quantification of transmigrated PBMC from the endothelial channel into the matrix. *n* > 3 devices. (C). Quantification of Th17 cells in the matrix that transmigrated from the endothelial channel. *n* = 3–6 devices. *P* values shown on the graphs.

It has been postulated that certain chemokines and cytokines, especially CCL20, attract T helper 17 (Th17) lymphocytes to damaged bile ducts in various liver diseases [20, 22]. Bacterial infections are frequently detected around the portal tracts of PSC patients and elevated serum endotoxin can increase the expression of adhesion molecules such as VCAM-1 and ICAM-1 [22–24]. To recapitulate inflammatory responses in PSC, we stimulated PSC cholangiocytes with IL-17A (50 ng ml^−1^ for 48 h) to generate an inflammatory phenotype. Simultaneously, we stimulated control endothelial cells in the same devices with LPS (1 *μ*g ml^−1^ for 4 h) to mimic bacterial infection and promote immune cell adhesion. LPS stimulation increased the expression of ICAM-1 and VCAM-1 on endothelial cells, as shown by immunofluorescence staining (figure S5). To investigate whether IL-17A-stimulated PSC cholangiocytes can attract Th17 cells, CD4 T cells were isolated from human peripheral blood and differentiated *in vitro* (figure S6), as previously described [25, 26]. Fluorescently-labeled human PBMC or differentiated CD4 T cells were then perfused into the endothelial channels and incubated overnight. PBMC and differentiated CD4 T cells adhered to endothelial cells and transmigrated into the surrounding matrix in all channels equally, regardless of LPS stimulation (figure 7). The number of transmigrated PBMC and differentiated CD4 T cells did not increase significantly under LPS stimulation but, notably, transmigration increased 18.6-fold (PBMC) and 3.5-fold (differentiated CD4 T cells) when cholangiocytes in the second channel had been stimulated with IL-17A. (Transmigration from the vascular channel to the matrix displayed no directional preference towards the biliary channel, likely because the chemoattractant cytokine can easily and rapidly diffuse all through the matrix around the vascular channel). These findings demonstrated that the VBDOC can successfully model immune cell recruitment.

## 4. Discussion

In this study we demonstrated that a VBDOC recapitulated vascularized bile ducts structurally and functionally by incorporating both organoid and organ-on-a-chip technology. Focusing on features of cholestatic liver diseases, we integrated several critical elements, including tubular structures, multicellular components, fibrous matrix, and mechanical stimuli into the VBDOC. The VBDOC can be used to study responses to mechanical forces, inflammatory responses including cytokine secretion, and immune cell recruitment. Furthermore, we demonstrated that the VBDOC can be constructed with cholangiocyte organoids derived from bile, which significantly broadens the cell source to include liver tissue explants, post-surgical biopsies, common bile duct brushings, and induced pluripotent stem cells.

While cholangiocyte organoids are used widely for the study of several cholangiopathies, organoids with only a single cell type do not capture the interactions between cholangiocytes and different cell types. Vascularized liver organoids have been developed by co-culturing iPSC-derived hepatic progenitor cells, mesenchymal cells and endothelial cells to study the inter-lineage interactions during early disease development [27, 28]; however, although these organoids formed bile canaliculi, the random organization of the vasculature, lack of bona-fide bile ducts and inability to perfuse either vessels or ducts has limited their application in the study of cholangiopathies.

Here we demonstrate that the VBDOC can support the coculture of endothelial cells, fibroblasts and cholangiocytes in independent but communicating compartments to model the structure and function of the vascular-biliary interface and enables independent treatment of vascular and biliary cells in the channels. Unlike tubular models with cholangiocytes that grow on a collagen-coated polyethersulfone hollow-fiber membrane with reversed polarity [29, 30], the VBDOC consists of a polarized bile duct within a natural scaffold containing mesenchymal cells, adjacent to a 3D vascular vessel that is cultured in endothelial medium. Small amounts of medium can be collected from endothelial and biliary reservoir ports separately for cytokine assays. The accessible lumen provides a tractable tool for studying cholangiocyte-bile interactions.

In addition, the VBDOC is a potential tool to study the role of mechanosensing organelles such as primary cilia. Open endothelial cell and cholangiocyte channels provide the chance to incorporate mechanical stimuli such as flow into the model and thereby study diseases like ciliopathies. By comparing static and flow conditions, we demonstrated that endothelial cells respond to shear flow as expected by aligning with the direction of flow and decreasing permeability, whereas neither was observed with cholangiocytes. The separation between the channels allows collection of medium from each channel for independent analyses and the application of distinct mechanical and biochemical stimuli to each channel, mimicking distinct physiological microenvironments and enabling study of the contribution of mechanical force and biochemical factors to pathology.

Organ-on-a-chip technology is often limited by cell availability and the need to choose between cell lines with poor fidelity and primary cells in limited supply. Integrating organoid and organ-on-a-chip technology has recently emerged as a superior, synergistic strategy because it enables long term propagation of primary cells while still retaining their physiological or pathological features [14, 31]. We demonstrated that the VBDOC can be constructed with cholangiocyte organoids from human tissue and bile. These cholangiocytes, when cultured in the device, display a biliary phenotype, with polarity and transport and barrier functions. Moreover, consistent with observations from PSC organoids [11], cholangiocytes in the VBDOC maintained the ability to secrete proinflammatory cytokines, including CCL20, in response to stimulation by IL-17.

In addition, by perfusing immune cells through the vascular channel, we modeled the recruitment of immune cells such as PBMC and differentiated Th17 cells to the bile ducts. In biopsies from patients with PSC, cell types including neutrophil and macrophages were found in the portal areas [5]. Neutrophil chemoattractants CXCL1 and CXCL5 were upregulated in the cytokine array experiments, consistent with previous experiments in which murine cholangiocytes were treated with IL-17A [32]. Macrophage chemoattractants such as MCP-1 were not detected, potentially because IL-17A stimulation alone is not enough to induce the secretion [16]. In the future, various combinations of cell types and stimulants including cytokines and bile acids could be tested to determine their contribution in the biliary inflammatory milieu. Thus, the VBDOC could be a valuable platform to study inflammatory and immune-mediated cholangiopathies.

VBDOC devices have limitations due to the nature of the technology. Their fabrication is labor intensive and thus high-throughput experiments are difficult. Additionally, the relatively low number of cells in the device makes it hard to perform traditional experiments like Western blotting. Small *in vivo* ducts (several microns in diameter) are currently not possible to replicate because of difficulty generating and seeding the channels. The VBDOC used HUVECs instead of liver sinusoidal endothelial cells or hepatic endothelial cells due to limited sources of cells and dedifferentiation; this may not accurately represent *in vivo* physiology. Despite these limitations, the VBDOC provides many benefits over conventional methods and has the potential to be a valuable tool to carry out mechanistic research on cholangiopathies.

## Data Availability

All data that support the findings of this study are included within the article (and any supplementary files).
